# Suspected anemia caused by maternal anti-Jra antibodies: a case report

**DOI:** 10.1186/s40364-015-0048-x

**Published:** 2015-08-21

**Authors:** Yasufumi Endo, Shoichi Ito, Yoshiko Ogiyama

**Affiliations:** Department of Pediatrics, Tendo City Hospital, 2-1 Gochome, Eki-nishi Tendo, Yamagata prefecture 994-0047 Japan; Akaishidai Children’s Clinic, 1-20 Rokuchome Akaishidai, Tomiya town, Miyagi prefecture 981-3332 Japan; Japanese Red Cross Tohoku Block Blood Center, 6-1 Nichome Akedori, Izumiku Sendai city, 981-3206 Miyagi prefecture Japan

**Keywords:** Irregular antibodies, Anti-Jra, ATP-binding cassette, Transporter, ATP-binding cassette G2 [ABCG2(CD338)]

## Abstract

Most cases of hemolytic disease of the newborn associated with anti-Jra are mild. However, rare cases of hydrops fetalis and severe anemia have been reported. We treated a neonate with anemia who was born with maternal anti-Jra, which were detected in the umbilical cord plasma. The Jra antigens in the neonate core blood red blood cells (RBCs) exhibited extremely weak reactivity to PEG-IAT, an anti-Jra reagent. However, upon re-examination of Jra antigen using PEG-IAT at 3 months postpartum, positivity was observed. Thereafter, upon performing PCR-SSP analysis of blood relatives targeting *ABCG2* at positions 376 and 421, we found that the mother was Jr(a−) with 376 T homozygosity, whereas the father was Jr(a+) with 376 C homozygosity and a carrier of a 421 C > A mutation. The first sibling, like the propositus, was Jr(a+), exhibiting 376 CT heterozygosity. However, the first sibling carried a 421 C > A mutation, whereas the propositus had no mutation at position 421. Setting the normal Jra (a+) type (376 C, 421 C) to 100 %, we identified the amount of Jra in RBC using FCM to be 82 % in the father, 31 % in the first sibling, and 69 % in the propositus. Furthermore, upon comparing peripheral blood and myelograms of the neonate at the time of birth, we found a low myeloid cells/erythroid cells ratio, undifferentiated erythroblasts, and reduced megakaryocytes. On the basis of these findings, we suggest that cell surface antigen is involved in the HDN caused by anti-Jra, and that a cytodifferentiation abnormality is present in the hematopoietic system.

## Background

More than 300 known blood group antigens are present in RBC, of which Jra is present at a very high frequency. An antibody directed to the Jra was first described in 1970 by Stroup and MacIlroy [1]. In Japan, there is a relatively high frequency of Jr(a−) individuals; approximately 0.06 % [2, 3]. In 2012, it was discovered that the ATP-binding cassette G2 (ABCG2/CD338) in the ATP-binding cassette transporter family carries the Jra, and the Jra (JR1) was adopted as the 32^nd^ human blood group system by the International Society for Blood Transfusions (ISBT) [4–7]. The incidence of hemolytic disease of the newborn due to anti-Jra is low, with majority of cases being mild. However, a couple of cases have been reported in which the patient developed hydrops fetalis [8–11]. In the present study, we treated a newborn who was hospitalized for transient tachypnea and presented with anemia and leukocytosis, which was believed to be associated with the presence of anti-Jra. To determine the cause, we assessed the amount of Jra and *ABCG2* in the RBC.

## Case presentation

### Perinatal and family history

The mother was gravida 5 para 2, including three miscarriages. On examination of irregular antibodies at 16 weeks of pregnancy with the first child (G4P1), the mother was Jr(a−) and had anti-Jra (antibody titer of 1:512). When pregnant with the second child (G5P2), the mother had an anti-Jra antibody titer of 1:64 at 20 weeks of pregnancy, and subsequently 1:256 at 27 weeks (IgG1 subclass). No other antibodies against blood group antigens were identified.

The first child was a girl, delivered at 36 weeks and 3 days of gestational age by Cesarean section at a different hospital due to breech presentation. The characteristics of the neonate included a birth weight of 2590 g, a height of 44.0 cm, a chest circumference of 32.0 cm, a head circumference of 34.0 cm, Apgar scores of 8 points at 1 min and 10 points at 5 min, and a placental weight of 560 g.

At 2 days of age, blood sampling was performed on suspicion of hyperbilirubinemia due to anti Jra, revealing a total bilirubin level of 9.3 mg/dL, with an unconjugated bilirubin level of 0.39 μg/dL. Therefore, the newborn was discharged from the hospital without phototherapy.

The second child was a girl delivered at 37 weeks and 6 days of gestational age, with a birth weight of 2808 g, a height of 49.0 cm, a head circumference of 32.5 cm, a chest circumference of 32.0 cm, Apgar scores of 7 points at 1 min and 8 points at 5 min, and a placental weight of 755 g.

From 35 weeks and 5 days of gestational age, the mother was administered ritodrine hydrochloride at a dose of 200 μg/min upon diagnosis of threatened premature delivery, and the baby was delivered by Cesarean section. Tachypnea and expiratory grunting were observed at birth, and with a SpO2 of 80 % persisting with room air, the baby was hospitalized.

The neonate was characterized by absence of bulging anterior fontanel, pallid skin, absence of cyanosis, grunting on chest auscultation, tachypnea, soft abdomen, and regular bowel sounds. Reduced translucency and partial dilatation were observed on chest radiography, and the neonate was diagnosed with transient tachypnea of newborn. After hospital admission, oxygen within the incubator was kept below 40 %, which improved grunting and reduced the respiratory rate. Furthermore, oxygen therapy was slowly decreased, and discontinued at 1 day of age. Blood sampling at the time of hospital admission revealed a WBC count of 31,500/μL (segmented neutrophils, 61.8 %; lymphocytes, 28.0 %; monocytes, 7.5 %; eosinophils, 1.8 %; basophils, 0.9 %); RBC, 2.20 × 10^6^/μL; Hb, 8.4 g/dL; Hct, 25.8 %; MCV, 117.3 fl; MCH, 38.2 pg; MCHC, 32.6 g/dL; Plt, 297 × 10^3^/μL; reticulocytes, 80.9 %; T-bil, 1.9 mg/dL; D-bil, 0.7 mg/dL; LDH, 355 IU/L; AST, 23I U/L; ALT, 8 IU/L; BUN, 7.9 mg/dL; Creat, 0.54 mg/dL; CPK, 92 IU/L; UA, 7.0 mg/dL; Na, 140.4 mEq/L; K, 4.82 mEq/L; Cl, 105.8 mEq/L; Ca, 10.4 mg/dL; IP, 5.3 mg/dL; Fe, 140 μg/dL; CRP, 0.30 mg/dL; IgM, 7 mg/dL; haptoglobin < 10, and ferritin, 255 ng/mL.

Examination for irregular cord blood antibodies revealed anti-Jra (antibody titer of 1:8); meanwhile, no other irregular antibodies were observed. The results of direct anti-globulin testing were negative. Upon examination using a 20 % PEG-IAT, cord RBC and maternal plasma reactivity were negative, but PEG-IAT with anti-Jra reagent revealed very weak binding. Thus, we refrained from determining the Jra type.

Blood sampled at 6 h and 24 h postpartum revealed bilirubin levels of 2.9 mg/dL and 2.8 mg/dL respectively, indicating no increase, and thereby phototherapy and exchange transfusions were not performed. Thereafter, hyperbilirubinemia was not observed, and at 13 days of age, the infant was discharged from the hospital.

**Fig. 1:**
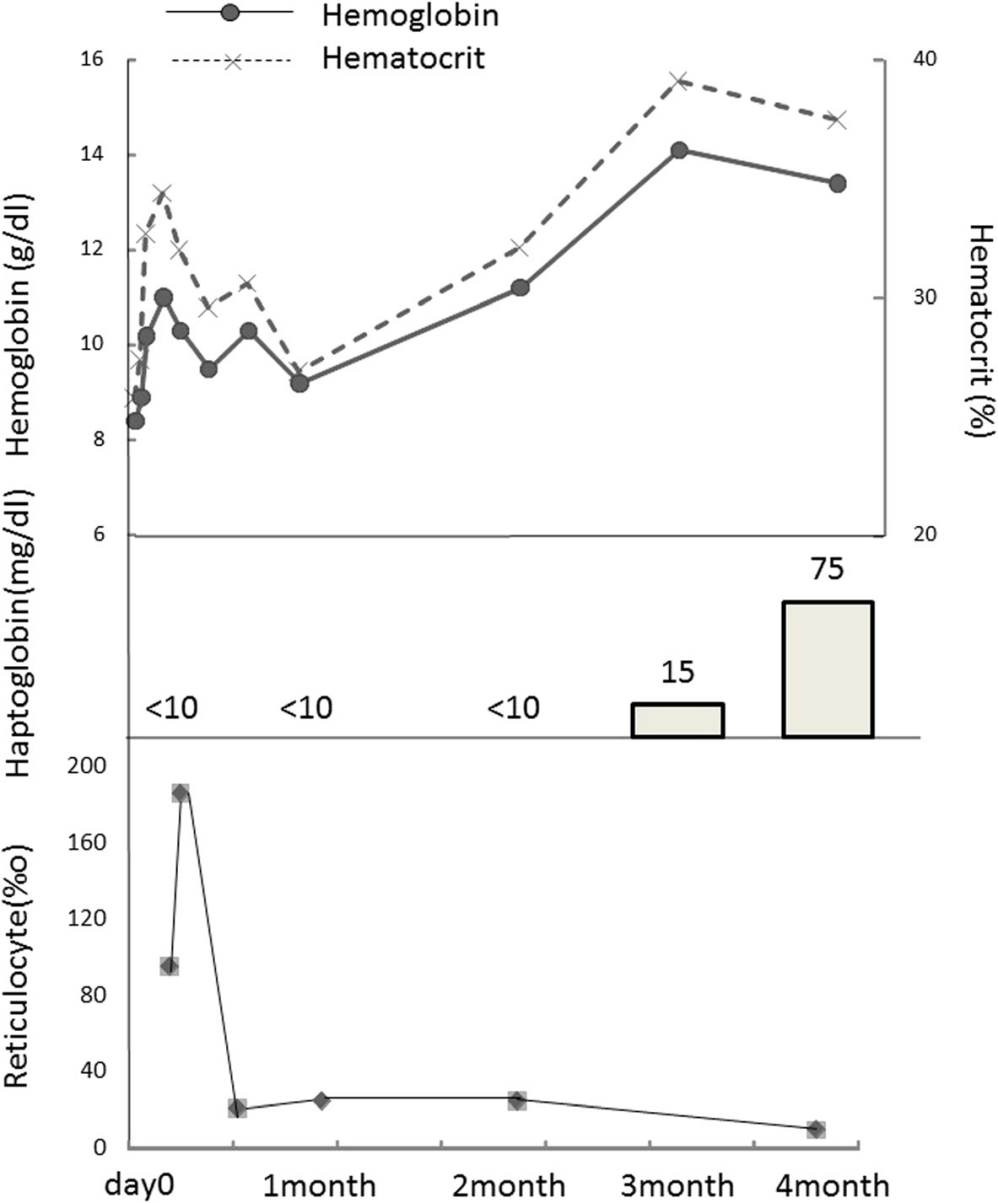
Changes in hemoglobin, hematocrit, haptoglobin, and reticulocytes from birth to 4 months postpartum. Hemoglobin and hematocrit levels as well as haptoglobin were increased after 3 months

Following discharge, we observed an increase of Hb to 14.1 g/dL and Hct to 39.1 % at three months. Haptoglobin was <10 mg/dL during treatment, but haptoglobin 2–2 type increased at 3 months (Fig. 1). Upon re-examination by PEG-IAT at 3 months using the anti-Jra, the infant’s RBC exhibited normal reactivity, and Jr(a+) was determined.

With consent from the family and after obtaining approval from the ethics committee of our hospital (Tendo Hospital notification 75) for familial blood group testing and genetic testing, we requested the Tohoku Block Blood Center to carry out the following analyses.

### Samples and methods

We evaluated the presence of Jra antigen in RBC in a test tube using three types of monoclonal anti-Jra (HIRO-133, HIRO-157, and HIRO-248) manufactured by the Red Cross. Further analyses were performed by FCM. The FACSCulibur (Becton, Japan ®) was used for FCM. High-titer, low-avidity (HTLA) anti-Jra were observed, and therefore, to enhance RBC antigen-antibody reactions, RBC underwent enzymatic treatment with 1 % ficin (MP Biomedicals ®). Primary antibodies were sensitized at 37 °C for 60 min, after which secondary antibodies (anti-human IgG: Fluorescein Labelled F [ab’]2) to human IgG (H + L) CODE:BI 4115 (Abliance®) were diluted 1:20 in PB, and sensitized at 4 °C for 60 min.

Genomic DNA was extracted from whole blood using DNA Blood Mini Kit (QIAamp Qiagen®, Chuo-ku Japan) according to the manufacturer’s instruction. For PCT, we used the Gene Amp PCR System 9700 series (Biosystems®). Gene analysis was performed by multiplex-PCR with our uniquely designed primers to detect SNPs in *ABCG2* at positions 34, 337, 376, 421, and 1515 (Table 1). The PCR-1 to PCR-8 included initial denaturation at 94 °C for 3 min and 35 cycles of denaturation(94 °C, 15 s) and annealing-extension(59 °C 1 min), followed by final extension at 72 °C for 5 min. PCR conditions of the PCR9-14 included initial denaturation at 94 °C for 3 min and 35 cycles of denaturation(94 °C, 15 s) and annealing-extension(60 °C 1 min), followed by final extension at 72 °C for 5 min using DNA polymerase(PrimeSTAR GXL, Takara Bio, Inc.). The obtained amplicons were subsequently sequenced using a cycle sequencing kit (BigDye Terminator, Version 1.1, Applied Biosystems Carlsbad, CA) and genetic analyzer (Model 3130xl, Applied Biosystems). After electrophoresis of the PCR product for 25 min at 200 V using a 7.5 % gel, the product was stained for 10 min with a solution containing 5 μL of ethidium bromide per 100 mL of deionized water, and the presence or absence of each specific band was evaluated.

**Table 1:** Oligonucleotide primers used for PCR

PCR (target)	Primer name	Nucleotide sequence (5′–3′)
JRA337/376/1515-SSP		
PCR-1 (Exon 13)	JRA1515-F4	AAGTCAAAGGCAGATGCTTC
PCR-2 (Exon 13)	JRA1515-R2	CCTTATCAGAGCAAACAGAG
PCR-3 (Exon 4)	JRA376-F4	CCAAGTGGATTATCTGGAGATG
PCR-4 (Exon 4)	JRA376-R6	TTGTCTCCTTTGTCTTTTACCAAACCCACTAATACTTTCTTG
PCR-5 (Exon 4)	JRA376-R1	CAAACCCACTAATACTTACTTA
PCR-6 (Exon 4)	JRA337-R10	TACATTTGAAATTGCCAGGTCA
PCR-7 (Exon 4)	GPC4-3	TGTGGCATTGTATCTTGTCCT
PCR-8 (Exon 4)	GPC4-4	GTGGCTATCTCGGGAAGAAT
JRA34/421-SSP		
PCR-9 (Exon 2)	JRA34-F2	TGGTATGGGCCATTCATTG
PCR-10 (Exon 2)	JRA34-R7	CATTGGTGTTGCCTCGTGACAT
PCR-11 (Exon 2)	JRA34-R10	CTTCGACAGCGCCCCTTCGGATTGGTGTTGCCTCGTGACAC
PCR-12 (Exon 5)	JRA421-F4	CTGACAGTGAGAGAAAACTTAA
PCR-13 (Exon 5)	JRA421-15	ACTACAACACTACCCGTGAGTGACGGTGAGCGAAAACTTAC
PCR-14 (Exon 5)	JRA421-R2	CACTTTATCCAGACCTAACTC-3′

## Results

### Family blood groups

The mother’s blood group was type O, D + C + c-E-e +, M-N + S-s +, P1−, Fy(a + b−), Jk(a + b +), Di(a–b+), Jr(a−); the father’s blood group was type A, D + C + c-E-e +, M-N + S-s +, P1+, Fy(a + b+), Jk(a + b+), Di(a–b +), Jr (a+); the first child’s blood group was type A, D + C + c-E-e +, M-N + S-s +, P1-, Fy(a + b−), Jk(a + b+), Di(a–b+), Jr(a+); and the proband’s blood group was type O, + C + c-E-e +, M–N + S-s +,P1+, Fy(a + b−), Jk(a–b+), Di(a–b+), Jr(a+).

### *ABCG2* (JR gene) analysis by PCR-SSP (Fig. 2)

**Fig. 2:**
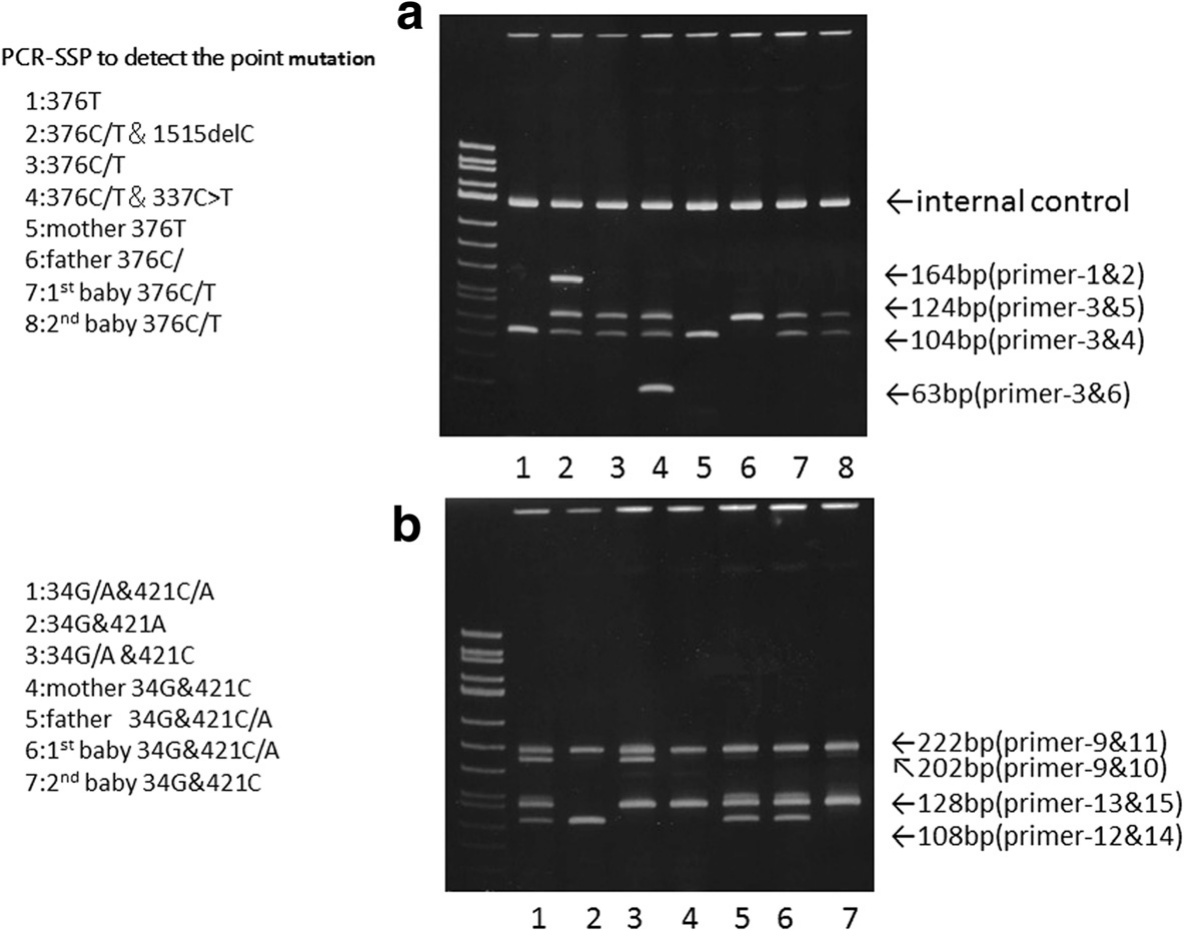
*ABCG2* analysis of the family by PCR-SSP. **a** 1: Jr(a−) phenotype 376 T/T, 2: Jr(a−) phenotype 376C/T & 1515delC, 3: Jr(a−) or Jr(a+) phenotype 376C/T, 4: Jr(a−) phenotype 376C/T& 337C > T, 5: mother 376 T/T, 6: father 376C/C, 7: 1^st^ baby 376C/T, 8: 2^nd^ baby 376C/T. **b** 1: 34G/A 421C/A, 2: 34G/G 421A/A, 3: 34G/A 421C/C, 4: mother 34G/G 421C/C, 5: father 34G/G 421C/A, 6: 1^st^ baby 34G/G 421C/A, 7: 2^nd^ baby 34G/G 421C/C

Genomic DNA was extracted from the peripheral blood of family members, and PCR-SSP was used to examine the presence or absence of genetic base substitutions at positions 376 and 421, which are primarily believed to affect Jra antigen density. The mother had Jr(a−) phenotype, exhibiting homozygous substitution of cytosine (C)—the common base at position 376 —for thymine (T) (376C > T), whereas no base mutations were observed at position 421. The father was homozygous for wild type 376 C/C, with a genetic mutation at position 421 C > A. The first child was heterozygous for 376 C/T with a 421 C > A mutation. The second child was heterozygous for 376 C/T, and unlike the first child, exhibited no mutation at position 421 (421 C/C).

### Comparison of the red blood cell antigen density by FCM

**Fig. 3:**
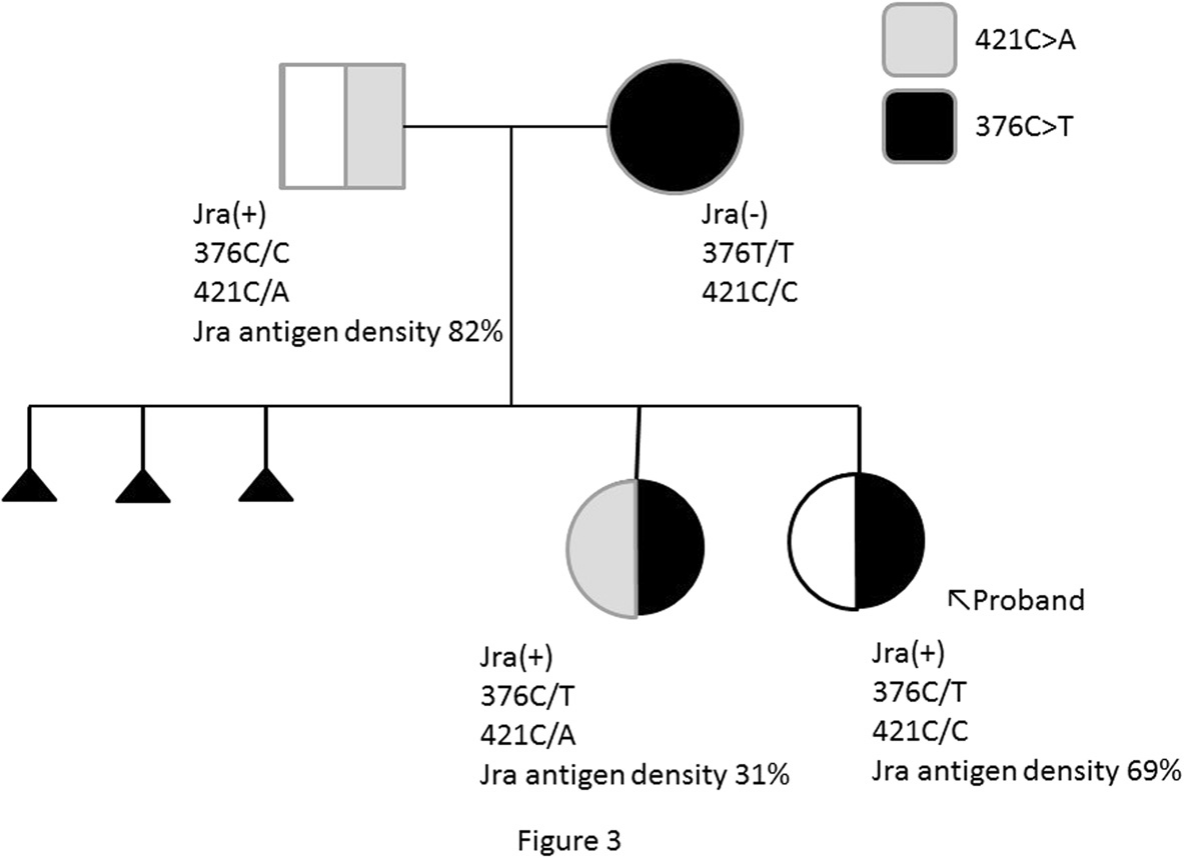
Jra antigen analysis by flow cytometry and family lineage. Jra antigen density was analyzed by flow cytometry, and the percentage of each antigen density was shown with 376 C/C and 421C/C defined as 100 %

Jra antigen densities of family members were compared according to mean fluorescence intensity. When compared with the mean fluorescence intensity of normal Jr(a+) (genotype: 376C/C, 421C/C) RBC set to 100 %, the mean intensity was 82 % for the father, 31 % for the first child, and 69 % for the second child (Fig. 3).

### Comparison of peripheral blood and bone marrow aspirate images taken immediately postpartum and at 4 months postpartum (Fig. 4)

**Fig. 4:**
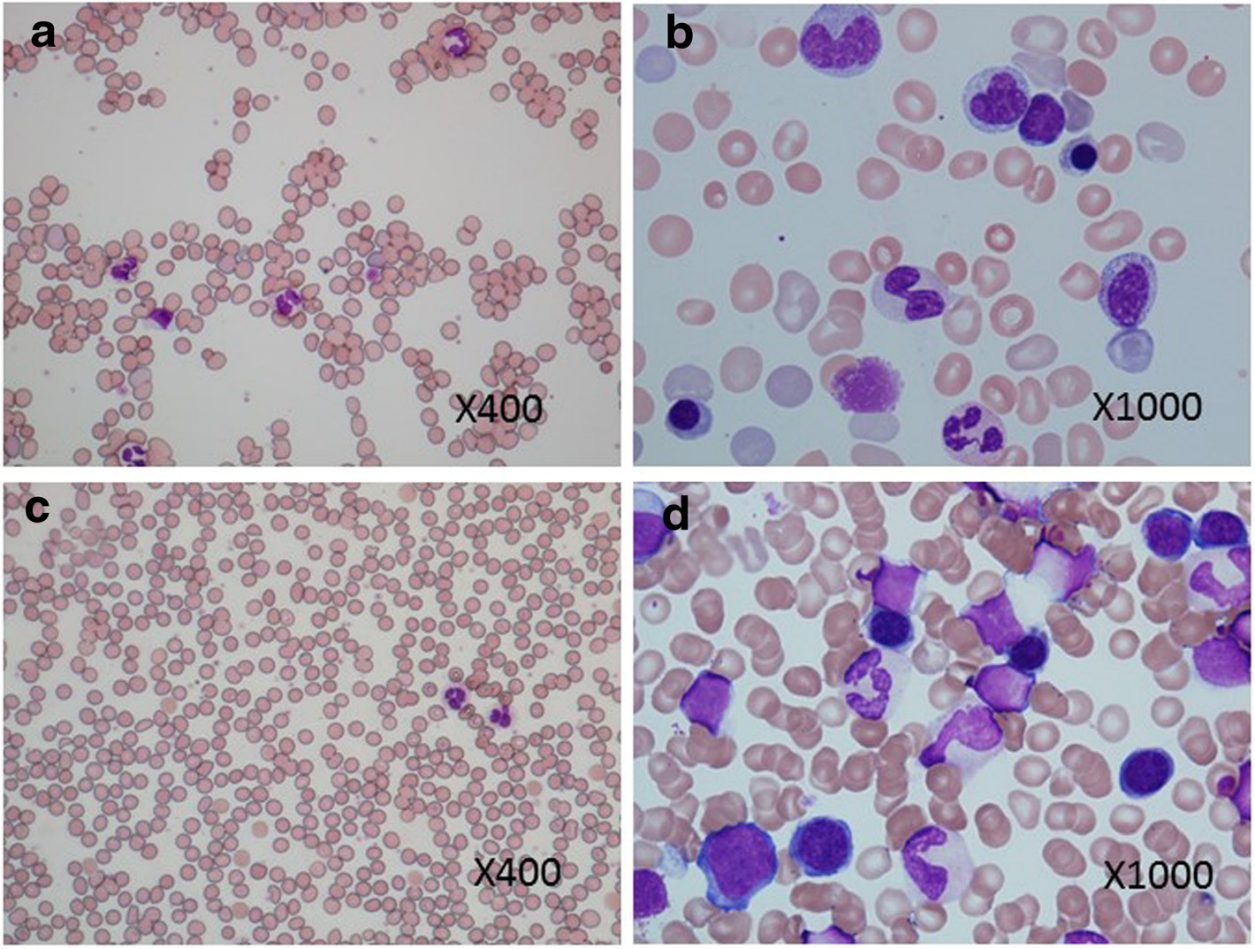
Peripheral blood smear (**a**) and bone marrow fluid smear (**c**) at 3 days of age. Peripheral blood showed a few hypersegmented neutrophils and megathrombocytes. Hyperplastic bone marrow was observed; however, no immature erythroblasts or megakaryocytes were found. Peripheral blood smear (**b**) and bone marrow fluid smear (**d**) at 4 months of age. The hypersegmented neutrophils in peripheral blood disappeared, and no megathrombocytes were observed. In the bone marrow, strongly basophilic immature erythroblasts were observed, and megakaryocytes appeared normal. Both findings were observed using May-Giemsa staining

A peripheral blood smear taken at 3 days of age revealed numerous polychromatic erythrocytes, spherocytes, leptocytes, and poikilocytes. Hypersegmented neutrophils and megathrombocytes were also observed (Fig. 4a). A peripheral blood smear performed at 1 month revealed a decline in hypersegmented neutrophils and megathrombocytes; at 4 months, these had disappeared (Fig. 4c). A bone marrow aspiration performed at 5 days of age revealed a cell count of 100,000/μL (myeloblasts, 0.2 %; myelocytes, 4.2 %; metamyelocytes, 3.2 %; band cells, 8.6 %; segmented neutrophils, 16.4 %; eosinophils, 1.6 %; basophils, 0.2 %; lymphocytes, 10.6 %; monocytes, 3.2 %; polychromatophilic erythroblasts, 28.0 %; orthochromatic erythroblasts, 23.0 %; and granulocytic/erythroblastic cell ratio, 0.7 %) with bone marrow hyperplasia. No megakaryocytes or macroblasts were observed. Among normoblasts, an increase in polychromatic and orthochromatic erythroblasts was observed in the absence of basophilic erythroblasts (Fig. 4b). Re-examination by bone marrow aspiration at 4 months, revealed a cell count of 420,000/μL (myeloblasts, 1.6 %; promyelocytes, 3.4 %; myelocytes, 4.0 %; metamyelocytes, 10.2 %; band cells, 10.8 %; segmented neutrophils, 10.6 %; eosinophils, 1.0 %; basophils, 0 %; lymphocytes, 38.8 %; monocytes, 2.6 %; macroblasts, 0.6 %; basophilic erythroblasts, 0.2 %; polychromatophilic erythroblasts, 10.2 %; orthochromatic erythroblasts, 0 %; and granulocytic/erythroblastic cell ratio, 4.3 %), and megakaryocytes were present (Fig. 4d).

## Discussion

*ABCG2* that carries the Jra antigen is located on the long arm of chromosome 4 (4q22) [6, 7]. It has been confirmed that ABCG2 is overexpressed on the surface of the placental villi, and if the fetal blood group is Jr(a+), then anti-Jra can be readily stimulated in maternal serum during pregnancy. The placental weight and gestation age in weeks for both the first and second child were comparable, and therefore, it appears that anti-Jra had little impact on placenta. Cord blood and peripheral RBC from the second child typed Jr(a-) immediately postpartum as measured by PEG-IAT with anti-Jra. However, at 3 months of age, the results were positive. Furthermore, it is believed that haptoglobin is not affected by changes in RBC in neonates; however, in the present study, haptoglobin showed a low value of <10 from birth to 2 months postpartum, which normalized after 3 months. Maternally derived IgG has a half-life of 20 days, and the infant’s total IgG titer is lowest at 3–4 months. This suggests that many cells expressing Jra antigen were affected by maternally derived anti-Jra, and that cells with low Jra antigen density may have remained. Various SNPs within *ABCG2* that carry the Jra antigen have been reported [7, 12, 13], among which 376 C > T and 421 C > A mutations have been found to affect Jra antigen density in RBC. It is believed that upon identification of Jra(a−) and Jr(a + ^w^) phenotypes, attempting gene analysis of genetic bases at positions 376 and 421 is useful when comparing Jra antigen densities [14]. Hence, with the first child with low antigen density being asymptomatic and the second child with high antigen density presenting with symptoms, this demonstrates that RBC antigen density is involved in the manifestation of symptoms. Upon bone marrow examination of the second child at 5 days and 4 months of age, the myeloid cells/erythroid cells ratio increased from 0.7 to 4.3 %. Furthermore, at 5 days of age, erythroblast subsets exhibited an extremely high proportion of polychromatic to orthochromatic erythroblasts, with no immature macroblasts being observed. Megakaryocytes also showed a considerable decrease at 5 days of age. Peripheral blood at 5 days of age showed megathrombocytes and hypersegmented leukocytes. It has recently been indicated that ABCG2 is responsible for maintaining the undifferentiated state in hematopoietic stem cells [14, 15]. While undifferentiated hematopoietic stem cells frequently express ABCG2, most precursor cells that have differentiated, exhibit decreased levels of ABCG2 expression, and expression is increased in erythroblastic cell differentiation. Therefore, it is believed that changes in the bone marrow and peripheral blood inhibited ABCG2 expression by anti-Jra antibodies, which may have led to excessive differentiation.

At present, anti-Kell are known to cause HDN from hematopoietic cellular dysfunction. Immature erythroblasts in the fetal liver are damaged by phagocytes, and therefore in this type of HDN, hemoglobin levels are not maintained in RBC, and hyperbilirubinemia is not present postpartum or in the cord blood [16]. ABCG2 is believed to protect cells from damage by heme by binding to heme in an anaerobic state [17]. This function is consequently inhibited by anti-Jra and may lead to anemia, in which no increase in hemoglobin is observed. Furthermore, as ABCG2 is thought to regulate cellular differentiation, anti-Jra may be involved in abnormal differentiation.

## Conclusion

 On the basis of the present study, we found that infants can exhibit RBC with considerably low Jra antigen density immediately postpartum, that Jra antigen density RBC may be involved in aggravation of the condition, and that anti-Jra may inhibit hematogenesis.

## Consent

Written informed consent was obtained from the patient’s legal guardians for publication of this case report and any accompanying images. A copy of the written consent is available for review by the Editor-in-Chief of this journal.

## Abbreviations

PCR-SSP: Polymerase chain reaction-sequence-specific primer
PEG-IAT: Polyethylene glycol-indirect antiglobulin test
FCM: Flow cytometry
